# Clinical and Genomic Epidemiology of Carbapenem-Nonsusceptible *Citrobacter* spp. at a Tertiary Health Care Center over 2 Decades

**DOI:** 10.1128/JCM.00275-20

**Published:** 2020-08-24

**Authors:** Ahmed Babiker, Daniel R. Evans, Marissa P. Griffith, Christi L. McElheny, Mohamed Hassan, Lloyd G. Clarke, Roberta T. Mettus, Lee H. Harrison, Yohei Doi, Ryan K. Shields, Daria Van Tyne

**Affiliations:** a Division of Infectious Diseases, Department of Medicine, University of Pittsburgh, Pittsburgh, Pennsylvania, USA; b Microbial Genomic Epidemiology Laboratory, University of Pittsburgh School of Medicine, Pittsburgh, Pennsylvania, USA; c Division of General Internal Medicine, Department of Medicine, University of Pittsburgh Medical Center, Pittsburgh, Pennsylvania, USA; d Graduate School of Public Health, University of Pittsburgh, Pennsylvania, USA; University of Iowa College of Medicine

**Keywords:** carbapenem, carbapenemase, *Citrobacter*, multidrug resistance

## Abstract

Carbapenem-nonsusceptible *Citrobacter* spp. (CNSC) are increasingly recognized as health care-associated pathogens. Information regarding their clinical epidemiology, genetic diversity, and mechanisms of carbapenem resistance is lacking. We examined microbiology records of adult patients at the University of Pittsburgh Medical Center (UMPC) Presbyterian Hospital (PUH) from 2000 to 2018 for CNSC, as defined by ertapenem nonsusceptibility. Over this time frame, the proportion of CNSC increased from 4% to 10% (*P = *0.03), as did daily defined carbapenem doses/1,000 patient days (6.52 to 34.5; *R*^2^ = 0.831; *P < *0.001), which correlated with the observed increase in CNSC (lag = 0 years; *R*^2^ = 0.660). Twenty CNSC isolates from 19 patients at PUH and other UPMC hospitals were available for further analysis, including whole-genome short-read sequencing and additional antimicrobial susceptibility testing. Of the 19 patients, nearly all acquired CNSC in the health care setting and over half had polymicrobial cultures containing at least one other organism. Among the 20 CNSC isolates, Citrobacter freundii was the predominant species identified (60%). CNSC genomes were compared with genomes of carbapenem-susceptible *Citrobacter* spp. from UPMC and with other publicly available CNSC genomes. Isolates carrying genes encoding carbapenemases (*bla*_KPC-2,_ *bla*_KPC-3_, and *bla*_NDM-1_) were also long-read sequenced, and their carbapenemase-encoding plasmid sequences were compared with one another and with publicly available sequences. Phylogenetic analysis of 102 UPMC *Citrobacter* genomes showed that CNSC from our setting did not cluster together. Similarly, a global phylogeny of 64 CNSC genomes showed a diverse population structure. Our findings suggest that both local and global CNSC populations are genetically diverse and that CNSC harbor carbapenemase-encoding plasmids found in other *Enterobacterales*.

## Abstract (precis)

Carbapenem-nonsusceptible *Citrobacter* spp. (CNSC) are increasingly recognized as health care-associated pathogens. Information regarding their clinical epidemiology, genetic diversity, and mechanisms of carbapenem resistance is lacking. We examined microbiology records of adult patients at the University of Pittsburgh Medical Center (UMPC) Presbyterian Hospital (PUH) from 2000 to 2018 for CNSC, as defined by ertapenem nonsusceptibility. Over this time frame, the proportion of CNSC increased from 4% to 10% (*P = *0.

## INTRODUCTION

Carbapenem-resistant bacteria have become a major health concern worldwide (1). There are limited therapeutic options for treating infections caused by these multidrug-resistant organisms, resulting in greater morbidity and mortality than infections caused by susceptible organisms (2). Furthermore, multidrug-resistant infections place an additional economic burden on health care systems (3). In recognition of this threat, the treatment and control of carbapenem-resistant organisms have been prioritized by both the Centers for Disease Control and Prevention and the World Health Organization (4, 5).

The recent increase in infections caused by carbapenem-resistant organisms in the United States has been largely driven by the dissemination of plasmid-encoded carbapenemase genes, which are often carried by members of the *Enterobacterales*, particularly Klebsiella pneumoniae (6, 7). However, rates of carbapenem resistance in other bacterial species have also increased (8). Among them, carbapenem-nonsusceptible *Citrobacter* spp. (CNSC) have become increasingly recognized as health care-associated pathogens (9–13). CNSC isolates have been found to be both genotypically and phenotypically diverse (14, 15), and their resistance to carbapenems is frequently caused by plasmid-borne carbapenemase genes, which can be readily acquired through horizontal gene transfer (12, 16).

Information regarding the clinical epidemiology, genetic diversity, and mechanisms of carbapenem resistance among CNSC in the United States is currently limited to a small number of studies and very few isolates (9, 17–19). Here, we aimed to investigate the emergence of CNSC within our health care system using epidemiology and genomics approaches. We conducted a retrospective analysis of CNSC prevalence and carbapenem use over the last 2 decades and compared the genomes of CNSC isolates from our center with those of other local *Citrobacter* isolates, as well as with CNSC genomes sampled from around the globe. We found that while the CNSC sampled from our center are highly genetically diverse, their diversity is consistent with the local carbapenem-susceptible *Citrobacter* population, as well as with CNSC sampled elsewhere.

(Preliminary data included in this work were presented at the IDWeek 2019 conference [20].)

## MATERIALS AND METHODS

### Study design and isolate collection.

This study was conducted at the University of Pittsburgh Medical Center Presbyterian Hospital (UPMC-PUH), an adult medical and surgical tertiary-care hospital with 762 total beds, 150 critical care unit beds, more than 32,000 yearly inpatient admissions, and over 400 solid-organ transplants per year. CNSC isolates were collected from UPMC-PUH as well as other UPMC hospitals. Ethics approval for this study was obtained from the Institutional Review Board of the University of Pittsburgh.

To investigate CNSC epidemiology at UPMC-PUH, microbiology records of adult patients with a positive clinical culture for CNSC from 1 January 2000 to 31 December 2018 were evaluated. Cases were excluded from reinclusion within 90 days of any CNSC culture. Carbapenem nonsusceptibility was defined as nonsusceptibility to any carbapenem according to the 2017 Clinical and Laboratory Standards Institute (CLSI) interpretative criteria (21). Antibiotic consumption was measured by daily defined doses (DDDs) of any carbapenem (22). To further phenotype and genotype CNSC at UPMC, 20 available CNSC isolates from 19 patients collected between 2013 and 2019 were included. Isolates were considered community associated if the organism was isolated from a specimen collected within 72 h following hospital admission; isolates collected after 72 h were considered health care-associated (23). Clinical characteristics and outcomes of patients with CNSC isolates that underwent further characterization were collected through retrospective chart review. The primary clinical outcome was in-hospital mortality and/or transfer to hospice.

### CNSC isolate characterization.

Initial species assignment was performed using standard clinical microbiology laboratory methods and was confirmed or modified after whole-genome sequencing. Carbapenem nonsusceptibility was initially determined by standard clinical microbiology laboratory methods and was confirmed by the Kirby-Bauer disk diffusion method as per the 2017 CLSI interpretative criteria (21). Susceptibility to additional agents was determined by the broth microdilution method (21). The presence of carbapenemase enzyme activity was assessed by modified carbapenem inactivation method (mCIM) (24).

### Genome sequencing and analysis.

Genomic DNA was extracted from pure overnight cultures of single bacterial colonies using a Qiagen DNeasy blood and tissue kit according to the manufacturer’s instructions (Qiagen, Germantown, MD). Library construction and sequencing were conducted using the Illumina Nextera NGS library prep kit or the Illumina Nextera XT DNA library prep kit (Illumina, San Diego, CA). Libraries were sequenced on an Illumina NextSeq system with 150-bp paired-end reads or an Illumina MiSeq system with 300-bp paired-end reads. Isolates with suspected plasmid-encoded carbapenemases were sequenced with long-read technology on a MinION device (Oxford Nanopore Technologies, Oxford, United Kingdom). Long-read sequencing libraries were prepared and multiplexed using a rapid multiplex barcoding kit (Oxford Nanopore Technologies catalog no. SQK-RBK004) and sequenced on R9.4.1 flow cells. Base-calling on raw reads was performed using Guppy v2.3.1 (Oxford Nanopore Technologies, Oxford, United Kingdom), and hybrid assembly was performed with both short Illumina reads and long Oxford Nanopore reads using Unicycler v0.4.8beta (25).

Illumina reads were quality filtered and assembled *de novo* using SPAdes v3.11. Species were identified by Kraken and by performing pairwise comparisons of average nucleotide identity on the assembled genomes using fastANI using the many-to-many method (26). Assemblies were clustered using the hierarchy module of the python package SciPy by single linkage method and a distance criterion of 5% difference in average nucleotide identity. Multilocus sequence typing (MLST) was performed with the mlst tool at https://github.com/tseemann/mlst. Genomes were annotated using Prokka v1.13 (27). Core genes were defined using Roary v3.12.0 with a 90% sequence identity cutoff (28). A phylogenetic tree based on a core gene alignment containing 1,606 genes identified by Roary was generated using RAxML v8.2.11 (29) by running 1,000 bootstrap replicates under the generalized time-reversible model of evolution, a categorical model of rate heterogeneity (GTR-CAT), with Lewis correction for ascertainment bias. The tree was visualized and annotated using Interactive Tree of Life (iTOL) v4 (30). The genomes of closely related isolates were compared with one another with breseq (31). Antimicrobial resistance gene and plasmid content were assessed by BLASTn of assembled contigs against downloaded ResFinder and PlasmidFinder databases, with 80% sequence identity and an 80% sequence coverage cutoff (32). Virulence gene content was assessed using the VirulenceFinder web interface with default settings and the Escherichia coli database (33). Carbapenemase-encoding contigs resolved from hybrid assembly of CNSC genomes were annotated using Prokka v1.13 (27), and resistance genes were identified using the ResFinder web interface with default settings (32). Carbapenemase-encoding contigs were compared to one another and to plasmid sequences downloaded from the RefSeq database (*n* = 18,364) using BLASTn of assembled contigs (34, 35). RefSeq and CNSC contigs whose sequences yielded at least 90% coverage of one another in either search direction were aligned to one another using EasyFig (36). Sequences were mapped to the *ompC* and *ompF* porin gene sequences from Citrobacter freundii ATCC 8090 (GenBank accession no. CP049015.1) using Geneious v11.1.5 to assess putative loss-of-function mutations, such as those resulting in premature stop codons, frameshift mutations, or large deletions. Available CNSC genomes were downloaded from the GenBank or Sequence Read Archive repositories maintained by the National Center for Biotechnology Information (NCBI).

### Statistics.

The proportion of CNSC was measured by dividing the number of CNSC isolates by the total number of *Citrobacter* species isolates tested for carbapenem susceptibility each year. Carbapenem (ertapenem, doripenem, meropenem, and imipenem) DDDs were measured per year at UPMC-PUH (22). Changes in the rate of carbapenem-nonsusceptible pathogen isolation over time were measured by linear regression, and comparison with the rate of antibiotic DDDs per year was conducted with time series cross-correlation analysis. Categorical data were compared using the χ^2^ test. Statistical analyses were performed using Stata V15 (StataCorp, College Station, TX) and R V3.5.1 (37).

### Data availability.

Genome sequence data generated in this study have been deposited in SRA or GenBank with accession numbers listed in Tables S1, S2, and S3 in the supplemental material. Accession numbers for genomes newly sequenced for this study are SAMN14007636 to SAMN14007655, SRR11038037 to SRR11038052, and SAMN14082844 to SAMN14082856.

## RESULTS

### Clinical epidemiology of CNSC.

**FIG 1:**
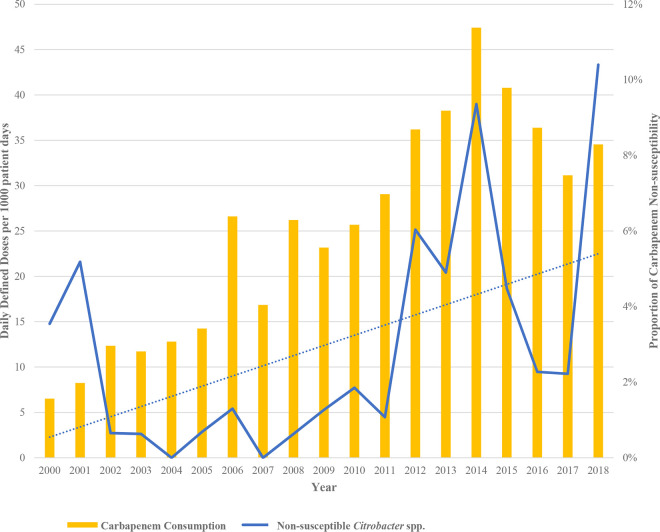
Carbapenem consumption and proportion of carbapenem-nonsusceptible *Citrobacter* spp. (CNSC), 2000 to 2018. Carbapenem daily defined doses (DDDs) per 1,000 patient days (yellow bars) and the proportion of *Citrobacter* species isolates that were carbapenem nonsusceptible (solid blue line) were quantified for each year between 2000 and 2018 at UPMC-PUH. Testing of 2,817 total *Citrobacter* species isolates revealed that 78 unique patients had CNSC (defined as ertapenem nonsusceptible). The dotted blue line shows a linear regression for increased CNSC proportion over time (*R*^2^ = 0.257; *P = *0.03). Carbapenem DDDs per 1,000 patient days also increased over time (*R*^2^ = 0.831; *P < *0.001) and correlated with the increase in CNSC (lag = 0 years; *R*^2^ = 0.660).

During the study period from 2000 through 2018, 78 unique patients with CNSC were identified from 2,817 *Citrobacter* isolates tested. *Citrobacter* spp. were the seventh most common carbapenem-nonsusceptible Gram-negative bacteria and fifth most common carbapenem-nonsusceptible *Enterobacterales* at our center during this time period. The proportion of *Citrobacter* species isolates that were CNSC increased significantly over time (*R*^2^ = 0.257; *P = *0.03), from 4% in 2000 to 10% in 2018 (Fig. 1). Daily defined doses (DDDs) of carbapenems per 1,000 patient days also increased during the same time period, from 6.52 in 2000 to 34.5 in 2018 (*R*^2^ = 0.831; *P < *0.001). We found that the increase in DDDs correlated with the increase in CNSC over the same period (lag = 0 years; *R*^2^ = 0.660) (Fig. 1).

### Isolation and characterization of CNSC.

**FIG 2:**
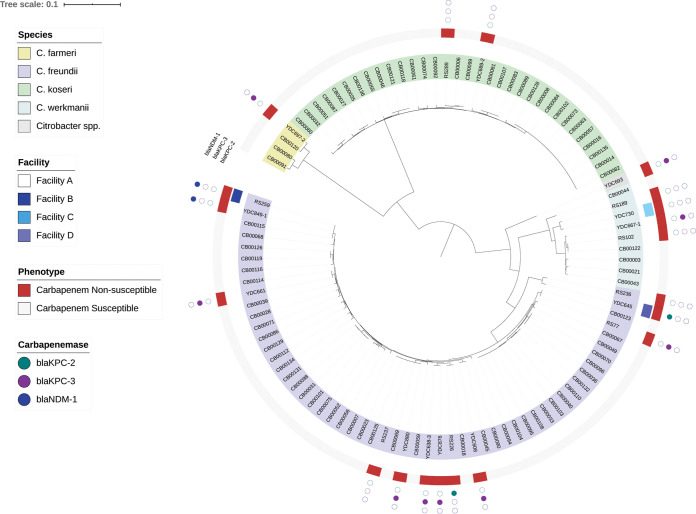
Local phylogeny of carbapenem-susceptible and -nonsusceptible *Citrobacter* spp. from UPMC. A phylogenetic tree of 102 local *Citrobacter* sp. genomes (82 carbapenem-susceptible and 20 carbapenem-nonsusceptible isolates) was generated based on an alignment of 1,606 core genes using RAxML (28). The tree was visualized and annotated using Interactive Tree of Life (iTOL) (29). The tree is annotated based on species, facility source, carbapenem susceptibility phenotype, and carbapenemase genes identified, if any, in the genome of each isolate.

Twenty CNSC isolates from 19 patients from UPMC-PUH and three additional UPMC hospitals were available for further analysis (Table 1). Among these patients, the median age was 65 (range, 26 to 92), and 37% were female (7/19). The majority of patients had multiple comorbidities, frequently acquired CNSC in the health care setting (84% [16/19]), had polymicrobial cultures (57% [11/19]), and had high rates of in-hospital mortality and discharge to hospice (47% [9/19]) (Table 1). We sequenced the genomes of all 20 CNSC isolates on the Illumina platform (Table S1) and constructed a phylogenetic tree that also included an additional 82 carbapenem-susceptible *Citrobacter* isolates collected by the Enhanced Detection System for Hospital-Associated Transmission (EDS-HAT) project (Fig. 2; Table S4) (38, 39). Among the 20 CNSC isolates, C. freundii was the predominant species (60% [12/20]), followed by Citrobacter werkmanii (20% [4/20]), C. koseri (10% [2/20]), and C. farmeri (5% [1/20]). One CNSC isolate, YDC693, was originally identified as C. freundii but showed only 90 to 92% average nucleotide identity to other C. freundii genomes (Table S4). This isolate appears to belong to a new, unnamed *Citrobacter* species. YDC693 (*Citrobacter* sp.) and YDC697-2 (*C. farmeri*) were cultured from the same patient and were from samples taken approximately 2 weeks apart. Their distribution throughout the genome phylogeny suggested that the CNSC isolates were largely genetically distinct from one another (Fig. 2; Table S4). The one exception was RS259 and YDC849-1, which were found to have fewer than 20 genetic variants (single nucleotide polymorphisms and insertion/deletion variants) that distinguished them from one another, despite being isolated from patients at two different facilities (facility A versus facility B).

**TABLE 1:** Clinical characteristics of patients with CNSC^*a*^

Isolate ID	Patient age	Gender	Yr	Source	Facility	CNSC	Culture site	Clinical syndrome	Polymicrobial culture	Other organism(s)	Comorbid condition(s)	Outcome of hospitalization
RS77	49	M	2018	Hospital	A	C. freundii	Rectal swab	Colonization	N	None	ESLD, liver transplant	Discharge to home
RS102	74	F	2018	Hospital	A	*C. werkmanii*	Rectal swab	Colonization	N	None	ESLD, liver transplant	In-hospital death
RS189	66	F	2017	Hospital	A	*C. werkmanii*	BAL fluid	Pneumonia	N	None	Diverticulosis	Discharge to facility
RS226	56	F	2018	Hospital	A	C. freundii	Urine	Colonization	Y	E. coli	Opiate dependency, CHF, HTN	In-hospital death
RS236	70	M	2018	Hospital	A	C. freundii	Peritoneal fluid	Intra-abdominal	Y	C. freundii (ESBL), Enterococcus faecium (VRE), Candida tropicalis, Candida parapsilosis	Pancreatic cancer, COPD, DM, HTN	Transfer to hospice
RS237	80	F	2018	Community	A	C. freundii	Urine	Colonization	N	None	Sinus OM	Discharge to home
RS259	61	F	2018	Hospital	B	C. freundii	Peritoneal fluid	Intra-abdominal	Y	E. coli, Pseudomonas aeruginosa	Metastatic lung cancer, COPD, DM	In-hospital death
RS289	92	M	2018	Community	A	*C. koseri*	Urine	UTI	Y	Enterococcus faecalis	Bladder cancer, dementia, CKD, COPD, CHF	Discharge to home
YDC608	57	M	2013	Hospital	A	C. freundii	BAL fluid	Colonization	N	None	HLD	Transfer to hospice
YDC638-2	27	M	2013	Hospital	A	C. freundii	Biliary drainage	Intra-abdominal	Y	E. faecium (VRE)	ESLD, Crohn’s diseases, PSC, liver transplant	Discharge to home
YDC645	67	F	2013	Hospital	C	C. freundii	Blood	SSTI/Endocarditis	Y	*Bacteroides* sp., Enterococcus raffinosus	CAD, CHF, DM, ESRD, dementia	Transfer to hospice
YDC661	64	M	2014	Hospital	A	C. freundii	BAL fluid	Pneumonia	Y	Stenotrophomonas maltophilia	Heart transplant	Discharge to facility
YDC667-1	73	M	2014	Hospital	A	*C. werkmanii*	BAL fluid	Pneumonia	Y	K. pneumoniae (ESBL)	CHF, DM, CAD, CKD	In-hospital death
YDC689-2	61	M	2015	Hospital	A	*C. koseri*	BAL fluid	Pneumonia	N	None	ESLD, liver transplant	Discharge to facility
YDC693^*b*^	65	M	2015	Hospital	A	*Citrobacter spp*.	BAL fluid	Pneumonia	Y	E. coli (ESBL)	SBT, adrenal insufficiency	In-hospital death
YDC697-2^*b*^	65	M	2015	Hospital	A	*C. farmeri*	Tracheostomy site drainage	SSTI	Y	Klebsiella oxytoca (KPC)	SBT, adrenal insufficiency	In-hospital death
YDC730	71	M	2015	Hospital	D	*C. werkmanii*	Pelvic abscess	Intra-abdominal	Y	E. faecium (VRE)	Multiple myeloma	Discharge home
YDC849-1	26	F	2018	Hospital	A	C. freundii	Urine	UTI	Y	C. freundii (NDM)	CVID	In-hospital death
YDC876	53	M	2019	Hospital	A	C. freundii	BAL fluid	Pneumonia	N	None	CAD, CHF	Discharge home
YDC880	73	M	2019	Hospital	A	C. freundii	Rectal swab	Colonization	N	None	Lung transplant	In-hospital death

a Abbreviations: BAL, bronchoalveolar lavage; CAD, coronary artery disease; CHF, congestive heart failure; CKD, chronic kidney disease; CNSC, carbapenem nonsusceptible *Citrobacter* spp.; COPD, chronic pulmonary disease; CVID, common variable immunodeficiency; DM, diabetes mellitus; ESBL, extended-spectrum beta-lactamase; ESLD, end-stage liver disease; ESRD, end-stage renal disease; F, female; HAP, hospital-acquired pneumonia; HCC, hepatocellular carcinoma; HLD, hyperlipidemia; HTN, hypertension; KPC, Klebsiella pneumoniae carbapenemase; M, male; NDM, New Delhi metallo-beta-lactamase; OM, osteomyelitis; PSC, primary sclerosing cholangitis; SBT, small bowel transplant; SSTI, skin and soft tissue infection; UTI, urinary tract infection; VRE, vancomycin-resistant *Enterococcus*.

b Same-patient isolates.

### Antimicrobial susceptibility and identification of antibiotic resistance and virulence genes.

While the CNSC isolates we collected were originally defined as nonsusceptible to ertapenem, they displayed variable patterns of susceptibility to other carbapenem antibiotics. Only about half of the isolates (55% [11/20]) were nonsusceptible to meropenem, with three isolates showing intermediate resistance and eight being resistant (Table 2). We tested all isolates for the presence of a carbapenemase using a modified carbapenem inactivation method (mCIM) (24), in which 13 isolates (65%) tested positive. Carbapenemase genes were present in the genomes of all 13 isolates (Table 2). Among the carbapenemases identified, *bla*_KPC-3_ was predominant (9/13), followed by *bla*_NDM-1_ (2/13) and *bla*_KPC-2_ (2/13). Analysis of the major porin-encoding genes revealed that *ompC* was intact in all CNSC isolates, while *ompF* was disrupted in at least six CNSC genomes (Table 2; Table S8). *ompF* disruption was not associated with increased meropenem MICs; however, we did not find evidence of *ompC* or *ompF* disruptions in any of the carbapenem-susceptible isolates. Next, we tested all 20 CNSC isolates against novel β-lactam–β-lactamase inhibitor agents and found that they were frequently susceptible to ceftazidime-avibactam (17/20 [85%]) and meropenem-vaborbactam (18/20 [90%]). As expected, both isolates with *bla*_NDM-1_ exhibited phenotypic resistance to both agents (Table 2). Isolate RS237 was also found to be resistant to ceftazidime-avibactam, even though its genome did not contain a carbapenemase sequence or evidence of porin mutations. We compared RS237 with the most closely related carbapenem-susceptible isolate, CB00023, and found a large number of mutations separating them from one another (Table S5). One of these was a missense mutation (S219I) in *acrE*, which is predicted to encode a multidrug export protein and could be a candidate resistance-associated gene.

**TABLE 2:** Carbapenemase genes, porin genotypes, antimicrobial susceptibilities, and mCIM results for 20 CNSC isolates^*a*^

Isolate	Organism	Carbapenemase gene	Porin genotype^*b*^		MIC (μg/ml) of^*c*^:			mCIM result
			*ompC*	*ompF*	Meropenem	Ceftazidime-avibactam	Meropenem-vaborbactam	
RS77	C. freundii	*bla*_KPC-3_	Intact	Intact	1	0.5	0.015	Positive
RS102	*C. werkmanii*		Intact	Intact	0.25	0.5	0.06	Negative
RS189	*C. werkmanii*		Intact	Intact	≤0.06	0.5	0.015	Negative
RS226	C. freundii	*bla*_KPC-2_	Intact	Disrupted	2 (I)	<0.25	0.015	Positive
RS236	C. freundii		Intact	Intact	2 (I)	2	0.5	Negative
RS237	C. freundii		Intact	Intact	4 (R)	64 (R)	0.5	Negative
RS259	C. freundii	*bla*_NDM-1_	Intact	Intact	16 (R)	>256 (R)	>8 (R)	Positive
RS289	*C. koseri*		Intact	Unknown	0.12	2	0.12	Negative
YDC608	C. freundii	*bla*_KPC-3_	Intact	Intact	16 (R)	4	0.06	Positive
YDC638-3	C. freundii	*bla*_KPC-3_	Intact	Disrupted	2 (I)	2	0.06	Positive
YDC645	C. freundii	*bla*_KPC-2_	Intact	Intact	≤0.06	<0.25	0.03	Positive
YDC661	C. freundii	*bla*_KPC-3_	Intact	Disrupted	1	4	0.06	Positive
YDC667-1	*C. werkmanii*	*bla*_KPC-3_	Intact	Disrupted	1	0.5	0.03	Positive
YDC689-2	*C. koseri*		Intact	Intact	0.5	4	0.12	Negative
YDC693^*d*^	*Citrobacter* sp.	*bla*_KPC-3_	Intact	Unknown	16 (R)	1	0.03	Positive
YDC697-2^*d*^	*C. farmeri*	*bla*_KPC-3_	Intact	Intact	32 (R)	4	0.12	Positive
YDC730	*C. werkmanii*		Intact	Intact	0.12	0.5	0.12	Negative
YDC849-1	C. freundii	*bla*_NDM-1_	Intact	Intact	16 (R)	>256 (R)	16 (R)	Positive
YDC876	C. freundii	*bla*_KPC-3_	Intact	Disrupted	8 (R)	1	0.06	Positive
YDC880	C. freundii	*bla*_KPC-3_	Intact	Disrupted	4 (R)	1	0.03	Positive

a All isolates were determined to be carbapenem nonsusceptible based on ertapenem nonsusceptibility.

b OmpC and OmpF porin genotypes were evaluated by comparison to C. freundii ATCC 8090. “Intact” indicates a protein sequence of expected length; “disrupted” indicates a premature stop codon, frameshift, or large deletion; “unknown” indicates that we were unable to assess the genotype due to a contig break (RS289) or highly divergent sequence (YDC693).

c Intermediate (I) and resistance (R) designations are based on CLSI breakpoints.

d Same-patient isolates.

In addition to carbapenemase genes, we also compared the non-β-lactam acquired antibiotic resistance gene content between CNSC and carbapenem-susceptible EDS-HAT isolates (Table S6). CNSC isolate genomes often carried genes encoding resistance to aminoglycoside, fluoroquinolone, and tetracycline antibiotic classes. Excluding β-lactam resistance genes, the average number of resistance genes was significantly higher among CNSC isolates than carbapenem-susceptible EDS-HAT isolates (mean [standard deviation], 6 [4] versus 2 [3]; *P < *0.001) (Table S6). Finally, we identified three CNSC isolates with virulence genes previously identified in E. coli (33). RS289 and YDC689-2 both carried a *senB* gene (GenBank accession no. AAZ89288), which encodes an enterotoxin (40). Additionally, YDC667-1 carried an *astA* gene (GenBank accession no. AF411067), which encodes an EAST-1 heat-stable toxin (41). These genes were also found among carbapenem-susceptible isolates (Table S7).

### Global phylogeny of CNSC.

**FIG 3:**
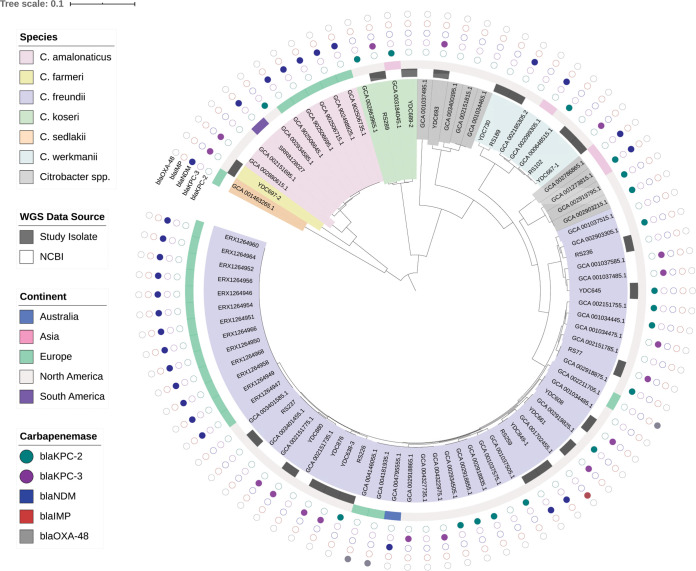
Global phylogeny of available CNSC genomes. A phylogenetic tree of 84 CNSC genomes (20 from this study and 64 from the NCBI) was generated based on an alignment of 1,842 core genes using RAxML (28). The tree was visualized and annotated using Interactive Tree of Life (iTOL) (29). The tree is annotated based on species, whole-genome sequencing data source, continent of isolation, and carbapenemase genes identified, if any, in the genome of each isolate.

To understand how the genomic diversity of the study isolates compared to that of CNSC isolates from other locations, we searched the NCBI databases for additional publicly available CNSC genomes. Using search terms “Citrobacter” and “carbapenem,” we identified 64 additional CNSC genomes (Table S3). A global phylogeny of these 64 CNSC genomes combined with the 20 from this study showed abundant genetic heterogeneity (Fig. 3). We investigated the species distribution of the global CNSC population using fastANI and found that, similar to our UPMC isolates, the global CNSC population was dominated by C. freundii (41/64 [64%]), followed by Citrobacter amalonaticus (9/64 [14%]), *C. werkmanii* (3/64, 5%), and *C. koseri* (2/64, 3%). Carbapenem-nonsusceptible *C. amalonaticus* was not found among our UPMC isolates, but it has been isolated in the United States, South America, and Europe (Fig. 3; Table S3). *Citrobacter* sp. YDC693 was found to cluster with an additional three isolates from the United States (Table S3). Three other global CNSC (two from the United States and one from China) appeared to belong to another distinct *Citrobacter* species with 90 to 93% average nucleotide identity to C. freundii. The proportion of global CNSC isolate genomes that encoded carbapenemases (49/64 [77%]) was similar to that of our UPMC isolate set (Fig. 3); however, the diversity of enzyme types was greater and included *bla*_NDM-5_, *bla*_IMP-38_, and *bla*_OXA-48_-like enzymes.

### Diversity of carbapenemase-encoding plasmids.

To better understand the genetic context of the carbapenemase enzymes encoded by our UPMC CNSC isolates, we conducted Oxford Nanopore long-read sequencing and hybrid assembly of the 13 carbapenemase-carrying isolate genomes (Table 3). A total of 11 complete, circular contigs were resolved from 10 isolates and nine patients, and all but one of these contigs contained replicons belonging to the IncA/C2, IncL/M, IncN, and unnamed RepA families. The genome assembly of RS259 contained a 21.4-kb circular contig encoding the *bla*_NDM-1_ carbapenemase on a class 1 integron, but it lacked readily identifiable plasmid replication machinery. This circular contig was highly similar to the *bla*_NDM-1_-encoding region of a 161-kb IncA/C2 plasmid resolved from the YDC849-1 genome. The RS259 genome appeared to also contain the remaining regions of the 161-kb plasmid from YDC849-1; however, the coverage was split across multiple contigs, suggesting excision of the class 1 integron into an unstable intermediate structure and/or issues with mobile element sequence assembly. Additionally, the YDC876 genome contained two plasmids of different sizes with distinct replicons that both harbored *bla*_KPC-3_ carbapenemase genes. Direct comparison of these two plasmids confirmed that they were distinct, even though they encoded the same carbapenemase and carried other acquired antimicrobial resistance genes (Table 3). Finally, three carbapenemase-encoding contigs were not completely resolved by hybrid assembly; in all cases, the contigs were short (less than 20 kb), and additional experiments would be needed to completely resolve their structures.

**TABLE 3:** Carbapenemase-encoding contigs identified in CNSC genomes

Isolate_contig	Length (bp)	Circular^*a*^	Replicon(s)	Carbapenemase gene	Carbapenemase-carrying element	Additional acquired antimicrobial resistance gene(s)^*b*^
RS77_21	10,011	No	None	*bla*_KPC-3_	Tn*4401b*-like	None
RS226_4	43,621	Yes	RepA	*bla*_KPC-2_	Tn*4401*-like	*bla*_TEM-1B_
RS259_9	21,420	Yes	None	*bla*_NDM-1_	Class 1 integron	*aac*(*3*)-*IId*, *aac*(*6*′)-*Ib-cr*, *aadA16*, *arr-3*, *catB3*, *dfrA27*, *mph*(A), *sul1*
YDC608_5	172,511	Yes	IncA/C2	*bla*_KPC-3_	Tn*4401b*-like	*aac*(*6*′)-*Ib*, *aadA1*, *ant*(*2*′')-*Ia*, *bla*_OXA-9_, *bla*_SHV-7_, *bla*_TEM-1A_
YDC638-3_3	213,257	Yes	IncA/C2	*bla*_KPC-3_	Tn*4401b*-like	*aac*(*6*′)-*Ib*, *aac*(*6*′)-*Ib-cr*, *aadA1*, *ant*(*2*”)-*Ia*, *bla*_OXA-9_, *bla*_SHV-7_, *bla*_TEM-1A_, *qnrA1*, *sul1*
YDC645_3	44,364	Yes	RepA	*bla*_KPC-2_	Tn*4401*-like	None
YDC661_9	15,031	No	None	*bla*_KPC-3_	Tn*4401b*-like	None
YDC667-1_41	4,894	No	None	*bla*_KPC-3_	Incomplete Tn*4401*	None
YDC693_4^*c*^	258,721	Yes	IncA/C2, IncN	*bla*_KPC-3_	Tn*4401*-like	*aac*(*6*′)-*Ib*, *aadA1*, *bla*_OXA-9_, *bla*_SHV-7_, *bla*_TEM-1A_, *dfrA14*
YDC697-2_6^*c*^	62,530	Yes	IncN	*bla*_KPC-3_	Tn*4401*-like	*dfrA14*
YDC849-1_2	160,983	Yes	IncA/C2	*bla*_NDM-1_	Class 1 integron	*aac*(*3*)-*Iid*, *aac*(*6*′)-*Ib-cr*, *aadA16*, *aph*(*3*′')-*Ib*, *aph*(*3*′)-*Ia*, *aph*(*6*)-*Id*, *arr-3*, *catB3*, *dfrA27*, *floR*, *mph*(A), *qnrS1*, *sul1*, *sul2*, *tet*(A)
YDC876_2^*d*^	176,497	Yes	IncA/C2	*bla*_KPC-3_	Tn*4401b*-like	*aac*(*6*′)-*Ib*, *aac*(*6*′)-*Ib-cr*, *aadA1*, *ant*(*2*′')-*Ia*, *bla*_OXA-9_, *bla*_SHV-7_, *bla*_TEM-1A_, *qnrA1*, *sul1*
YDC876_4^*d*^	78,220	Yes	IncL/M	*bla*_KPC-3_	Tn*4401b*-like	*aac*(*6*′)-*Ib*, *aac*(*6*′)-*Ib-cr*, *aadA1*, *bla*_OXA-9_, *bla*_TEM-1A_
YDC880_4	88,095	Yes	IncL/M	*bla*_KPC-3_	Tn*4401*-like	*aadA2*, *bla*_SHV-30_, *dfrA12*, *sul1*

a Circular contigs were identified through hybrid assembly with Unicycler (25).

b Antimicrobial resistance genes were identified by querying the ResFinder database (30).

c Contigs from same-patient isolates.

d Contigs from the same isolate.

**FIG 4:**
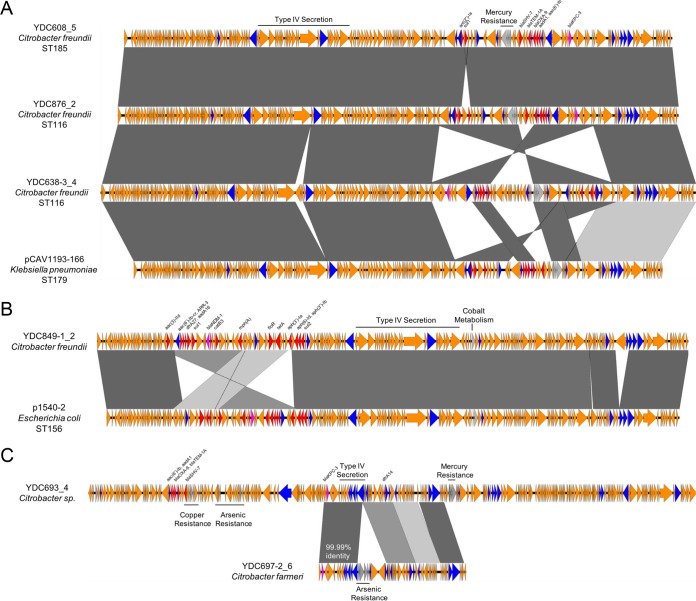
Carbapenemase-encoding plasmid diversity among and between CNSC genomes. CNSC genome contigs were compared to each other and to sequences deposited in the National Center for Biotechnology Information (NCBI). Sequences were aligned to one another with EasyFig. Sequence names are in the format “isolate_contig” based on hybrid assembly or correspond to the sequence name from NCBI. Bacterial species and sequence type are listed, where available. Open reading frames are colored by function (blue, mobilization; pink, carbapenemase; red, other antibiotic resistance; gray, metal interacting; orange, other/hypothetical). Antibiotic resistance genes, metal-interacting operons, and type IV secretion system components are labeled. Gray blocks between sequences indicate regions of >5 kb with >98% nucleotide identity, with darker shading indicating higher identity. Nucleotide identity between the *bla*_KPC-3_-encoding Tn*4401*-like regions of YDC693_4 and YDC697-2_6 (from two isolates of different *Citrobacter* species from the same patient) is noted with white text in panel C.

To determine how the carbapenemase-encoding plasmids in this study compared to one another and whether they were unique to our study, we conducted pairwise comparisons of the resolved plasmids. In addition, we searched the RefSeq database (35) for plasmids that showed substantial homology and high sequence identity to one or more of the CNSC plasmids from our UPMC isolates. One of the plasmids we resolved (RS226_4) matched a *bla*_KPC-2_-carrying plasmid from a publicly available C. freundii isolate genome (GenBank accession CP037739.1) with 100% coverage and 100% nucleotide identity, despite the isolates themselves being genetically distinct from one another. Three other plasmids that we identified (YDC608_5, YDC876_2, and YDC638-3) were highly similar to one another and were found in C. freundii isolates belonging to two different sequence types (ST185 and ST116) (Fig. 4A). While YDC608 and YDC876 belonged to two different C. freundii sequence types, their plasmids were more similar to one another than the plasmid from YDC638-3, which belonged to the same sequence type as YDC876. These plasmids were also highly similar to the pCAV1193-166 plasmid found in a *bla*_KPC_-carrying K. pneumoniae isolate from Virginia (Fig. 4A) (42). Separately, the YDC849-1_2 plasmid carrying *bla*_NDM-1_ had high similarity with plasmid p1540-2, which was found in a carbapenem-resistant E. coli isolate from Hong Kong (GenBank accession no. CP019053.1) (Fig. 4B). In addition, the *bla*_KPC-3_-carrying plasmids YDC693_4 and YDC697-2_6 were from isolates of different species that came from the same patient. Despite being different sizes (259 kb versus 63 kb), the plasmids showed some similarity to one another, and in particular, the Tn*4401*-like elements carrying *bla*_KPC-3_ on each plasmid contained only 1 mutation in more than 17 kb of sequence (Fig. 4C). These data suggest possible transfer of a *bla*_KPC-3_-encoding mobile element between the isolates from this patient; however, independent acquisition or independent transfer from another species cannot be ruled out.

## DISCUSSION

In this study, we conducted a retrospective review of the clinical and genomic epidemiology of CNSC over the past 2 decades at a large health care center in the United States. We analyzed the genomes of 20 CNSC and 82 carbapenem-susceptible *Citrobacter* spp. sampled locally, as well as 64 publicly available genomes sampled from around the globe. We found that the rates of CSNC had increased significantly over the last 2 decades at our center, that CSNC were frequently acquired in the health care setting along with other health care-associated organisms, and that patients from whom CNSC were isolated often had poor clinical outcomes. Our phylogenetic analyses revealed genetically diverse CNSC populations both locally and globally, suggesting that CNSC most often arise independently from one another. We also found that carbapenem nonsusceptibility was often mediated by acquisition of carbapenemase genes, with *bla*_KPC-3_ being the predominant carbapenemase identified among CNSC isolates in our setting.

*Citrobacter* spp. have become increasing recognized as a cause of multidrug-resistant health care-associated infections around the world (10, 43–45), and prior reports identified CNSC predominantly from health care sources and often in association with nosocomial outbreaks (9, 12, 46). We detected a significant increase in the proportion of *Citrobacter* isolates that were carbapenem nonsusceptible over the last 2 decades, which correlated with increased use of carbapenems at our center. While the incidence of carbapenem-resistant organisms has increased worldwide over recent years (7, 47), attention has been largely focused on other carbapenem-resistant members of the *Enterobacterales*, such as *Enterobacter* spp., E. coli, and *Klebsiella* spp. (7). As with other Gram-negative species, increasing antibiotic resistance among *Citrobacter* spp. is of significant concern, as our findings show that many CNSC appear to have acquired resistance genes from other bacteria by horizontal gene transfer.

Our analysis revealed extensive genomic diversity among both CNSC and carbapenem-susceptible *Citrobacter* isolates in samples from our center. This is similar to previous analyses of CNSC from non-U.S. centers that used more classical molecular typing methods (14, 48, 49). Among *Citrobacter* species, C. freundii is most commonly associated with both clinical disease (45) and multidrug-resistant phenotypes (14, 15, 50). Our findings were consistent with these prior reports; C. freundii was the most frequent CNSC species we observed, though we also found CNSC belonging to four additional species. The global CNSC population was similarly diverse, but C. freundii was again the most prevalent species observed, which may be due to its higher rate of antibiotic resistance compared to other *Citrobacter* spp. (44, 45).

The term “carbapenem nonsusceptibility” encompasses a wide range of phenotypic susceptibilities, which can be caused by different mechanisms. Among the CNSC isolates we collected, roughly two-thirds were found to produce carbapenemases, a rate that is similar to those in prior reports (14) and to that in the global CNSC population. CSNC have been found to encode a diverse array of carbapenemases. For example, a study by Arana et al. found five different carbapenemase types among *Citrobacter* species isolates collected from Spain (14), and similar results were also demonstrated in a study of carbapenemase-producing *Enterobacterales* in China (15). It has been suggested that carbapenemase diversity depends on local geography (47), and future studies of larger populations may confirm or refute this notion. The high diversity of carbapenemase-encoding plasmids we found, all from isolates of a single genus at a single hospital, highlights the complexity of antibiotic resistance gene transfer between pathogens in the hospital setting (51). Even with a relatively small number of isolates, we observed identical and closely related plasmids in genetically distinct bacteria, identical *bla*_KPC-3_-encoding mobile elements on different plasmids carried by the same bacterial isolate, and similar carbapenemase-encoding plasmids in CNSC of different species that were isolated from the same patient. These findings underscore the highly dynamic and variable transfer of carbapenemase-encoding mobile genetic elements into and among CNSC isolates.

There were several limitations to this study. While our study presents the largest genomic analysis of CNSC from the United States, the number of isolates we included was still rather limited. Moreover, the isolates we collected represent a convenience sample of available isolates, which may introduce bias. Furthermore, the correlation between carbapenem consumption and proportion of CNSC is a strictly ecologic analysis. Additionally, our genomic analysis of resistance determinants was limited to acquired carbapenemase genes, and we did not investigate other resistance mechanisms, such as chromosomal *ampC* genes and efflux pumps, or perform functional testing of outer membrane protein mutations, which are known to be associated with carbapenem resistance. Furthermore, many of the global isolate genomes we analyzed were from the United States and/or were part of outbreak investigations; thus, they may not be representative of the true global diversity of CNSC. Additionally, we made only *in silico*, sequence-based comparisons of the plasmids we resolved; as such, we cannot comment on their capacity for conjugative transfer. Finally, we were unable to determine whether the poor clinical outcomes among the patients from whom CNSC were isolated were indeed attributable to CNSC infection.

As CNSC become more prevalent in the health care system, further studies will be needed to increase our understanding of their genomic diversity and resistance mechanisms. In particular, examining the local and global epidemiology of horizontal transfer of drug resistance elements among *Citrobacter* spp. and between *Citrobacter* and other *Enterobacterales* species would provide valuable insights into risk factors and other trends that could be targeted to limit the occurrence and spread of CNSC.

## Supplementary Material

Supplemental file 1
